# Serum MicroRNAs as Potential Biomarkers for Early Diagnosis of Hepatitis C Virus-Related Hepatocellular Carcinoma in Egyptian Patients

**DOI:** 10.1371/journal.pone.0137706

**Published:** 2015-09-09

**Authors:** Tarek K. Motawi, Olfat G. Shaker, Shohda A. El-Maraghy, Mahmoud A. Senousy

**Affiliations:** 1 Biochemistry Department, Faculty of Pharmacy, Cairo University, Cairo, Egypt; 2 Medical Biochemistry and Molecular Biology Department, Faculty of Medicine, Cairo University, Cairo, Egypt; Drexel University College of Medicine, UNITED STATES

## Abstract

Circulating microRNAs are deregulated in liver fibrosis and hepatocellular carcinoma (HCC) and are candidate biomarkers. This study investigated the potential of serum microRNAs; miR-19a, miR-296, miR-130a, miR-195, miR-192, miR-34a, and miR-146a as early diagnostic biomarkers for hepatitis C virus (HCV)-related HCC. As how these microRNAs change during liver fibrosis progression is not clear, we explored their serum levels during fibrosis progression in HCV-associated chronic liver disease (CLD) and if they could serve as non-invasive biomarkers for fibrosis progression to HCC. 112 Egyptian HCV-HCC patients, 125 non-malignant HCV-CLD patients, and 42 healthy controls were included. CLD patients were subdivided according to Metavir fibrosis-scoring. Serum microRNAs were measured by qRT-PCR custom array. Serum microRNAs were deregulated in HCC versus controls, and except miR-130a, they were differentially expressed between HCC and CLD or late fibrosis (F3-F4) subgroup. Serum microRNAs were not significantly different between individual fibrosis-stages or between F1-F2 (early/moderate fibrosis) and F3-F4. Only miR-19a was significantly downregulated from liver fibrosis (F1-F3) to cirrhosis (F4) to HCC. Individual microRNAs discriminated HCC from controls, and except miR-130a, they distinguished HCC from CLD or F3-F4 patients by receiver-operating-characteristic analysis. Multivariate logistic analysis revealed a panel of four microRNAs (miR-19a, miR-195, miR-192, and miR-146a) with high diagnostic accuracy for HCC (AUC = 0.946). The microRNA panel also discriminated HCC from controls (AUC = 0.949), CLD (AUC = 0.945), and F3-F4 (AUC = 0.955). Studied microRNAs were positively correlated in HCC group. miR-19a and miR-34a were correlated with portal vein thrombosis and HCC staging scores, respectively. In conclusion, studied microRNAs, but not miR-130a, could serve as potential early biomarkers for HCC in high-risk groups, with miR-19a as a biomarker for liver fibrosis progression to cirrhosis to HCC. We identified a panel of four serum microRNAs with high accuracy in HCC diagnosis. Additional studies are required to confirm this panel and test its prognostic significance.

## Introduction

Hepatocellular carcinoma (HCC) is the third cancer-related cause of death worldwide, with chronic hepatitis C virus (HCV) infection as a major risk factor of HCC [1]. Noteworthy, HCC constitutes 13% of all cancers in Egypt and is the second most frequent cancer in men [2]. Chronic HCV accounted for 94% of HCC cases in Egypt in 2010, with 6000–7000 deaths/year due to HCC [3].

Low survival of HCC patients is attributed to late diagnosis, tumor recurrence, and metastasis, with novel biomarkers for early diagnosis urgently needed. Prognosis and survival rates are improved significantly with early diagnosis. Current diagnostic methods such as imaging techniques and serological tumor markers, alpha-fetoprotein (AFP), Lens culinaris agglutinin-reactive AFP, and des-γ-carboxy prothrombin are insufficient for early detection of HCC [4,5]. AFP was rejected for surveillance or diagnosis of HCC by the American Association for the Study of Liver Diseases guidelines (AASLD) (July 2010). MicroRNAs (miRNAs) negatively regulate gene expression and may act as oncogenes, or tumor suppressors, or play dual roles in hepatocarcinogenesis regulating cell cycle, cell proliferation, differentiation, migration, and apoptosis [6]. Indeed, miRNAs dysregulation during HCV infection has been linked with the initiation and progression of HCC [7,8]. Circulating miRNAs are deregulated in HCC and are emerging as novel stable and easily detectable biomarkers for early diagnosis of HCC [9]. Thereby, profiling of circulating HCC-related miRNAs may unravel new molecular biomarkers with high sensitivity and specificity for HCC.

HCC usually arises in the setting of cirrhosis or bridging fibrosis in HCV-associated chronic liver disease (CLD) [10,11]. HCC risk predictors that identify the subset of advanced fibrosis and cirrhosis patients with the highest risk of HCC are needed [12]. Accordingly, monitoring of fibrosis is mandatory as it reflects disease progression, ultimately to HCC. As liver biopsy for fibrosis staging presents invasiveness, sample variability, and other limitations, circulating miRNAs were proposed as novel non-invasive methods to assess histological disease severity in chronic HCV [13,14]. However, information about miRNAs correlation with fibrosis progression in CLD is limited. Few miRNAs, including serum miR-122, miR-34a, miR-20a, and miR-92a were associated with liver fibrosis progression in chronic HCV patients [13,14]. Therefore, monitoring of circulating miRNA signatures during liver fibrosis progression could be clinically relevant as a non-invasive diagnostic tool for early detection of HCC.

The present study aimed to investigate the expression profiles of selected miRNAs; miR-19a, miR-296, miR-130a, miR-195, miR-192, miR-34a, and miR-146a in sera from Egyptian patients with HCV-related HCC, their correlations with clinicopathological data, and their potential usefulness as biomarkers for early diagnosis of HCC. In addition, as how these miRNAs change during liver fibrosis progression is not clear, we explored their serum levels during fibrosis progression in non-malignant HCV-associated CLD and if they could potentially serve as non-invasive biomarkers for fibrosis progression to HCC. Our study revealed that these miRNAs, but not miR-130a, could serve as potential biomarkers for early detection of HCC in high-risk groups, with miR-19a as a biomarker for liver fibrosis progression to cirrhosis to HCC.

## Materials and Methods

### Patients

The study included 237 HCV-infected Egyptian patients; 112 HCV-related HCC patients and 125 non-malignant HCV-associated CLD patients, who were admitted to the outpatient’s clinic of the liver unit, Tropical Medicine Department, Kasr El-Aini Hospital, Cairo University, and Cairo Fatemic Hospital, Ministry of Health and Population, Cairo, Egypt. All patients were anti-HCV positive with detectable serum HCV RNA by PCR (TaqMan assay reagents and Ambion, the RNA Company-one step RT-PCR Kit, USA). All HCC patients were on top of HCV cirrhosis and HCC diagnosis was made upon the presence of hepatic focal lesions diagnosed by abdominal ultrasound and confirmed by computed tomography (CT) and/or magnetic resonance imaging according to European Association of the Study of the Liver (EASL) guidelines (2012). Liver disease severity in HCC patients was assessed by Child-Puph grade and HCC staging was done using Barcelona Clinic Liver Cancer (BCLC) staging system [15].

Diagnosis of HCV-associated CLD was based on standard clinical, biochemical, serological, and ultrasonographic criteria, as well as the histopathological data obtained at liver biopsy. Metavir scoring was used to stage fibrosis on a five-point scale: F0, no fibrosis; F1, portal fibrosis alone; F2, portal fibrosis with rare septae; F3, portal fibrosis with many septae (bridging fibrosis); and F4, cirrhosis [16]. All CLD patients were of significant fibrosis from F1 to F4, with no patients with F0 stage. CLD patients were further subdivided according to their fibrosis stage into 2 subgroups; early fibrosis (mild/moderate fibrosis; F1-F2, n = 75) and late fibrosis (severe fibrosis/cirrhosis; F3-F4, n = 50). F4 patients were Child A (n = 24) and Child B (n = 8). History taking, clinical examination, and routine laboratory investigations were made for every patient. Patients with chronic hepatitis B virus (HBV) or any other identifiable cause for chronic hepatitis other than HCV, any associated malignancies other than HCC, or who received previous treatment for HCC or antiviral therapy for HCV were excluded.

In addition, a total of 42 gender (males, n = 28 and females, n = 14) and age (mean±SD, 44±12.5) matched healthy volunteers were evaluated as controls; all have normal liver function tests, normal AFP levels (<10 ng/ml), normal hepatic ultrasound, and negative for hepatitis B surface antigen (HBsAg), hepatitis B core antibodies (HBc-Ab), and HCV RNA by PCR. Written informed consent was obtained from all patients and controls for gene analysis. The study protocol and informed consent were approved by the ethics committee of the Faculty of Pharmacy, Cairo University and conformed to the ethical guidelines of the Helsinki Declaration.

### Laboratory tests

Fasting venous blood samples were collected from all patients for routine workup, including complete blood picture, liver function tests, prothrombin concentration and prothrombin-international normalized ratio, AFP, anti-HCV titer, HBsAg, and HBc-Ab using commercially available assays.

### Rationale of miRNAs selection and serum miRNAs assay by qRT-PCR

We assayed serum samples for 7 miRNA candidates (miR-19a, miR-296, miR-130a, miR-195, miR-192, miR-34a, and miR-146a) having a known association with HCC. Each of these miRNAs have been linked mechanistically to HCC or have predicted oncogenic or tumor suppressor target genes [6,17–20]. miR-19a, a member of the angiogenic MYC-induced 17–92 miRNA cluster [20], targets PTEN, SOX4 [21], and the proapoptotic APAF1, Beclin1, and PAK6 [22]. miR-296 was linked to tumor angiogensis by regulating vascular endothelial growth factor (VEGF) receptors overexpression [23] and may also contribute to carcinogenesis by dysregulating p53 [24]. miR-130a was predicted to target genes involved in mitotic cell cycle, protein modification process, and cell differentiation [19]. miR-34a and miR-192 are p53-responsive tumor suppressor miRNAs that induce apoptosis and cell cycle arrest [6,18]. miR-34a was also predicted to target oncogenes such as PDGFRA, E2F5, Foxp1, c-Met, Wnt1, and Notch1 [6]. miR-195 is a tumor suppressor by regulating the G1/S transition in HCC [17] and targeting the oncogenic fibroblast growth factor 7 (FGF7) and growth hormone receptor (GHR) [6]. miR-146a was reported as a tumor suppressor and a negative regulator of toll-like receptor signaling and NF-κB via TRAF6 and IRAK-1 [25]. Importantly, miR-296 [26], miR-130a, miR-146a, miR-195 [27,28], and miR-34a [29–31] were deregulated in HCC tissues versus healthy livers. However, the invasive nature of liver biopsy may limit the use of these miRNAs as HCC biomarkers. In addition, compared with normal, we have previously demonstrated that serum levels of these 7 miRNAs were deregulated in chronic HCV patients; these miRNAs were interferon-related, and five of them were associated with the outcome of standard interferon therapy [32]. Moreover, serum miR-19a and miR-192 were also proposed as biomarkers for HBV-associated HCC [33]. Therefore, we hypothesized that these 7 serum miRNAs would represent a broad variety of functions in HCC and had high potential for release into the circulation. However, the clinical relevance of using these miRNAs as potential non-invasive serum biomarkers for HCV-related HCC detection is not yet known.

#### RNA extraction and reverse transcription

Total RNA, including miRNAs was extracted by miRNeasy extraction kit (Qiagen, Valencia, CA) using QIAzol lysis reagent according to the manufacturer’s instructions. The quality of RNA was determined using NanoDrop2000 (Thermo scientific, USA). Reverse transcription (RT) was carried out on 100 ng of total RNA in a final volume 20 μl RT reactions (incubated for 60 min at 37°C and 5 min at 95°C) using miScript II RT Kit (Qiagen, Valencia, CA) according to the manufacturer’s instructions.

#### Quantitative real-time PCR

Serum expression levels of mature miRNAs, hsa-miR-19a-3p, hsa-miR-296-5p, hsa-miR-130a-3p, hsa-miR-195-5p, hsa-miR-192-5p, hsa-miR-34a-5p, and hsa-miR-146a-5p was evaluated using miScript miRNA PCR custom array (Qiagen, Valencia, CA) according to the manufacturer’s protocol. The housekeeping miScript PCR control, miRNA SNORD68 was used as internal control, as earliarly reported [34,35]. Our previous experience also suggests that SNORD68 is a stable normalization control and possible for use in miRNA PCR analysis [32]. For real-time PCR of each miRNA, 2.5 μl diluted RT products was mixed with 5.5 μl RNase-free water, 10 μl QuantiTect SYBR Green PCR Master Mix and 2 μl miScript universal primer (reverse primer), and then added to a custom Rotor-Disc 100 miRNA PCR array which contains miRNA-specific miScript primer assays (Qiagen, Valencia, CA). The Rotor-Disc was sealed with optical thin wall strips. Real-time PCR was performed using Rotor gene Q Real-Time PCR System (Qiagen, Valencia, CA) with the following conditions: 95°C for 30 min, followed by 40 cycles at 94°C for 15 s, 55°C for 30 s, and 70°C for 30 s. The cycle threshold (Ct) is the number of cycles required for the fluorescent signal to cross the threshold in real-time PCR. Fold change of miRNA expression levels was calculated using the formula 2^-∆∆Ct^ using healthy controls as calibrator, where ∆∆Ct = [Ct (target, test)-Ct (reference, test)]-[Ct (target, calibrator)-Ct (reference, calibrator)] [36].

### Statistical analysis

Values were expressed as mean±standard deviation (SD), median (25%–75% percentiles) or number (percentage) when appropriate. Clinical data of three independent groups were compared using one way ANOVA and Tukey Kramer’s multiple comparisons test. AFP levels were log transformed to enable parametric statistical tests. Categorical data were compared by Chi square (*X* ^*2*^) test. The non-parametric Mann-Whitney *U*-test was used for comparison of miRNA data (2^-∆∆Ct^) from independent samples from 2 groups as miRNA data were not normally distributed. Serum miRNAs in individual fibrosis stages (F1 to F4) were compared by Kruskal-Wallis test. The diagnostic accuracy of miRNAs was evaluated by receiver-operating-characteristic (ROC) analysis and the area under the curve (AUC) was calculated. The end point is that miRNA could discriminate HCC from late fibrosis (F3-F4) subgroup. Logistic regression analysis was done to identify predictor miRNAs associated with the risk of HCC. Univariate analysis was conducted to determine the relationship between expression level of each miRNA and presence/absence of HCC (HCC vs. CLD + healthy controls). Significant predictor miRNAs in the univariate analysis were included in a stepwise forward multivariate analysis (*P*<0.05 for entering the model and *P*<0.1 for removal from the model) to determine the final predictor miRNAs for the probability of being diagnosed with HCC. The predicted probability of being diagnosed with HCC was used as a surrogate marker to construct the ROC curve. AUC was used as an accuracy index for evaluating the diagnostic performance of the selected miRNA panel. Correlations between parameters were determined by Spearman or Pearson correlation when appropriate. *P*<0.05 was considered significant, with a 95% confidence interval (CI). Statistical analyses were performed using computer program Statistical Package for the Social Science (SPSS, Chicago, IL) software version-15 for Microsoft Windows and GraphPad Prism-5.0 (GraphPad Software, CA).

## Results

### Demographic and clinical features of HCC and non-malignant CLD patients

The clinical features of HCC and non-malignant CLD patients are shown in Table 1. Studied patients showed a significant trend of elder age with progression of liver disease from F1-F2 to F3-F4 fibrosis to HCC (*P*<0.0001). Gender was not significantly different between studied groups (*P* = 0.79). However, there was a male predominance in HCV-related liver disease patients in the three groups representing 76%, 76%, and 82.14% in F1-F2, F3-F4, and HCC groups, respectively. Serum levels of AST, ALP, AFP, total bilirubin (*P*<0.0001, for each), and direct bilirubin (*P* = 0.0009) were significantly higher, whereas hemoglobin levels were significantly lower (*P*<0.0001) in HCC patients versus the other two groups. Serum ALT was only significantly different between HCC and F1-F2 group (*P* = 0.001). Hepatic synthetic functions; albumin and prothrombin concentration tended to decrease significantly (*P* = 0.0001, *P*<0.0001, respectively) during liver disease progression among studied groups. HCC and F3-F4 patients showed significantly lower platelet count versus F1-F2 patients. Total leucocyte count was not significantly different between studied groups (*P* = 0.46).

**Table 1:** Demographic and clinical data of HCC and non-malignant chronic liver disease patients.

Parameter	Non-malignant CLD (n = 125)		HCC (n = 112)	*P* value
	F1+F2 (n = 75)	F3+F4 (n = 50)		
Age (years)	42±9.2^A^	48±5.5^B^	60±8.3^C^	<0.0001
Gender				0.79
Male, n (%)	57 (76)	38 (76)	92 (82.14)	
Female, n (%)	18 (24)	12 (24)	20 (17.86)	
ALT (IU/l)	55±24.6^A^	60.5±24.8^AB^	80.4±47.3^B^	0.001
AST(IU/I)	50.39±20^A^	65.28±28.1^A^	118.3±55.2^B^	<0.0001
ALP (IU/l)	104.3±42.4^A^	115±68.9^A^	169.5±60^B^	<0.0001
Total bilirubin (mg/dl)	0.72±0.22^A^	0.87±0.34^A^	1.8±1.4^B^	<0.0001
Direct bilirubin (mg/dl)	0.42±0.19^A^	0.45±0.14^A^	0.8±0.85^B^	0.0009
Albumin (g/dl)	4.3±0.5^A^	3.7±0.8^B^	3.18±0.4^C^	0.0001
Prothrombin concentration %	93±6.7^A^	82.6±5^B^	68.9±16.3^C^	<0.0001
AFP Log 10 (ng/ml)	0.57±0.4^A^	0.81±0.2^A^	2.6±1.17^B^	<0.0001
Hemoglobin (g/dl)	14.2±1.4^A^	13.2±1.8^A^	11.7±2.3^B^	<0.0001
Total leukocyte count (x10^3^/mm^3^)	6.4±2.26^A^	6.3±2.32^A^	5.8±2.6^A^	0.46
Platelet count (x10^3^/mm^3^)	224.5±24.6^A^	166.2±41.6^B^	127.2±76.5^B^	<0.0001

Values are expressed as mean±SD or number (percentage). Clinical data were analyzed by ANOVA and Tukey Kramer’s multiple comparison test. Gender was analyzed by *X* ^*2*^ test. Groups with different letters show significant difference (*P*<0.05), while those with same letters show no significant difference (*P*>0.05). ALT: alanine aminotransferase; AST: aspartate aminotransferase; ALP: alkaline phosphatase; AFP: alpha-fetoprotein.

### Clinicopathological characteristics of HCC patients

The clinicopathological characteristics of HCC patients are shown in Table 2. HCC patients produced a wide range of AFP values from normal to 46870 ng/ml (mean±SD, 4405±10470 ng/ml). Normal AFP levels (up to 10 ng/ml) are present in as many as 14% of patients, with 75% having AFP >20 ng/ml and 46% with AFP>400 ng/ml at time of diagnosis. 92.86% of HCC patients had Child-Pugh grade A and B, 75% had performance status 0, and 71.43% had stage B in BCLC staging. Regarding CT imaging, hepatic focal lesions were single in 50% of patients, arising from the right lobe (64.3%), 57.15% were of size >5cm, with 21.43% of patients having portal vein (PV) thrombosis.

**Table 2:** Clinicopathological characteristics of HCC patients.

Parameter	Number (%)
AFP level	
<20 ng/ml	28 (25)
20–400 ng/ml	32 (28.6)
>400 ng/ml	52 (46.4)
Child-Pugh grade	
A	64 (57.14)
B	40 (35.72)
C	8 (7.14)
Performance status (PS)	
PS 0	84 (75)
PS1-2	20 (17.86)
PS>2	8 (7.14)
BCLC score	
Stage 0	0 (0)
Stage A	4 (3.57)
Stage B	80 (71.43)
Stage C	20 (17.86)
Stage D	8 (7.14)
Number of focal lesions	
Single	56 (50)
Multiple	56 (50)
Site of focal lesions	
Right lobe	72 (64.3)
Left lobe	20 (17.85)
Both	20 (17.85)
Tumor size by CT	
<3 cm	4 (3.57)
3–5 cm	44 (39.28)
>5 cm	64 (57.15)
Portal vein thrombosis	
Yes	24 (21.43)
NO	88 (78.57)

BCLC: Barcelona Clinic Liver Cancer staging, CT: computed tomography.

### Differential expression of serum miRNA levels in HCC patients

**Fig 1:**
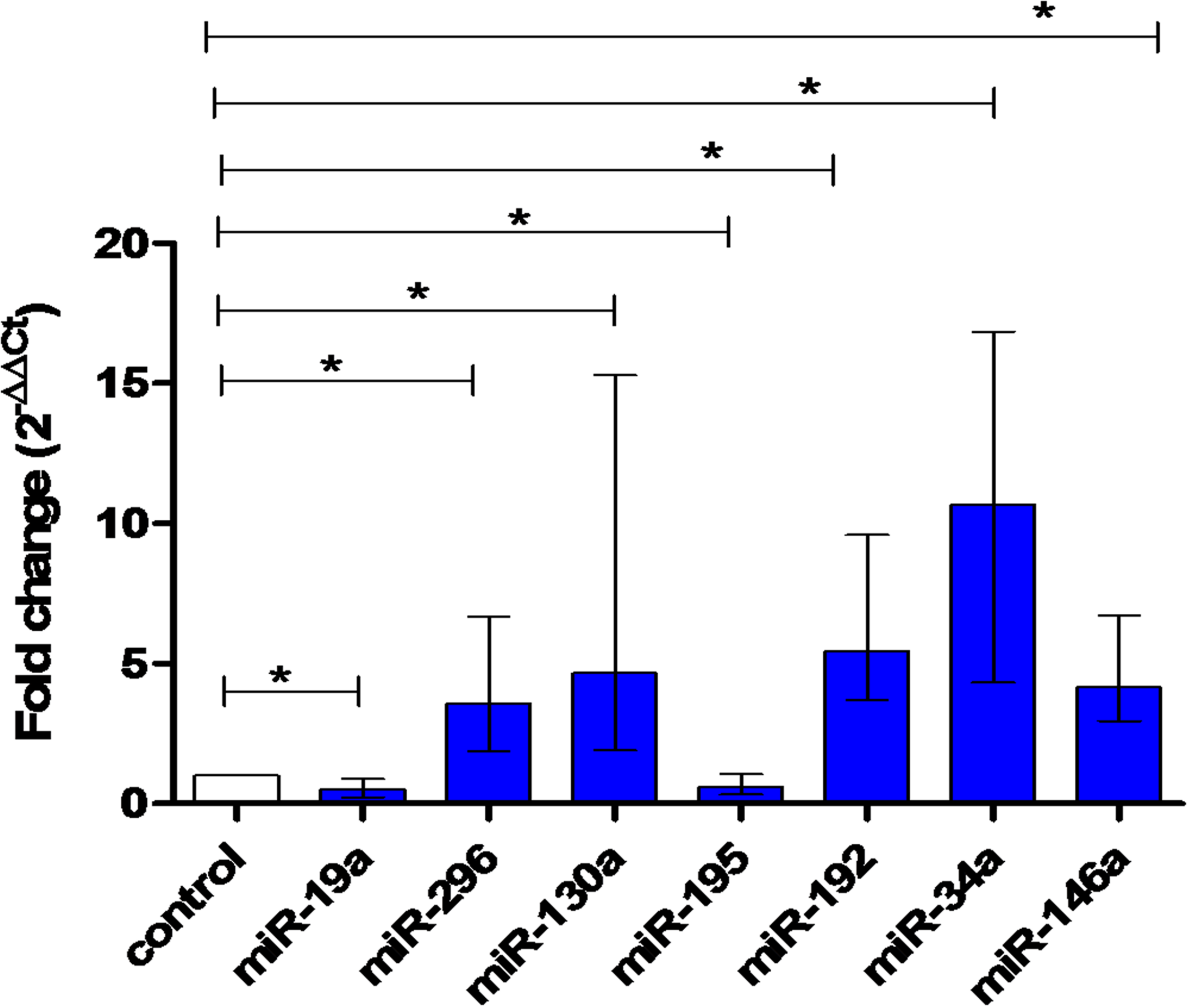
Differential expression of serum miRNA levels in HCC and healthy controls. Relative expression of miRNAs miR-19a (*P* = 0.0002), miR-296 (*P*<0.0001), miR-130a (*P*<0.0001), miR-195 (*P* = 0.04), miR-192 (*P*<0.0001), miR-34a (*P*<0.0001), and miR-146a (*P*<0.0001) in serum of HCC (n = 112) compared to healthy controls (n = 42). Data are presented as median with interquartile range. Data were analyzed by Mann-Whitney *U*-test.* means statistical significance (*P*<0.05).

We examined serum miRNA profiles in HCC group comparing to healthy controls or CLD patients using Mann-Whitney *U*-test. Comparing to healthy controls, miR-296, miR-130a, miR-192, miR-34a, and miR-146a (*P*<0.0001, for each) were upregulated in HCC patients (median fold change, 3.16, 4.65, 5.54, 11.19, and 4.16, respectively), whereas miR-19a and miR-195 were downregulated in HCC group (median fold change, 0.51 (*P* = 0.0002) and 0.62 (*P* = 0.04), respectively) (Fig 1).

**Fig 2:**
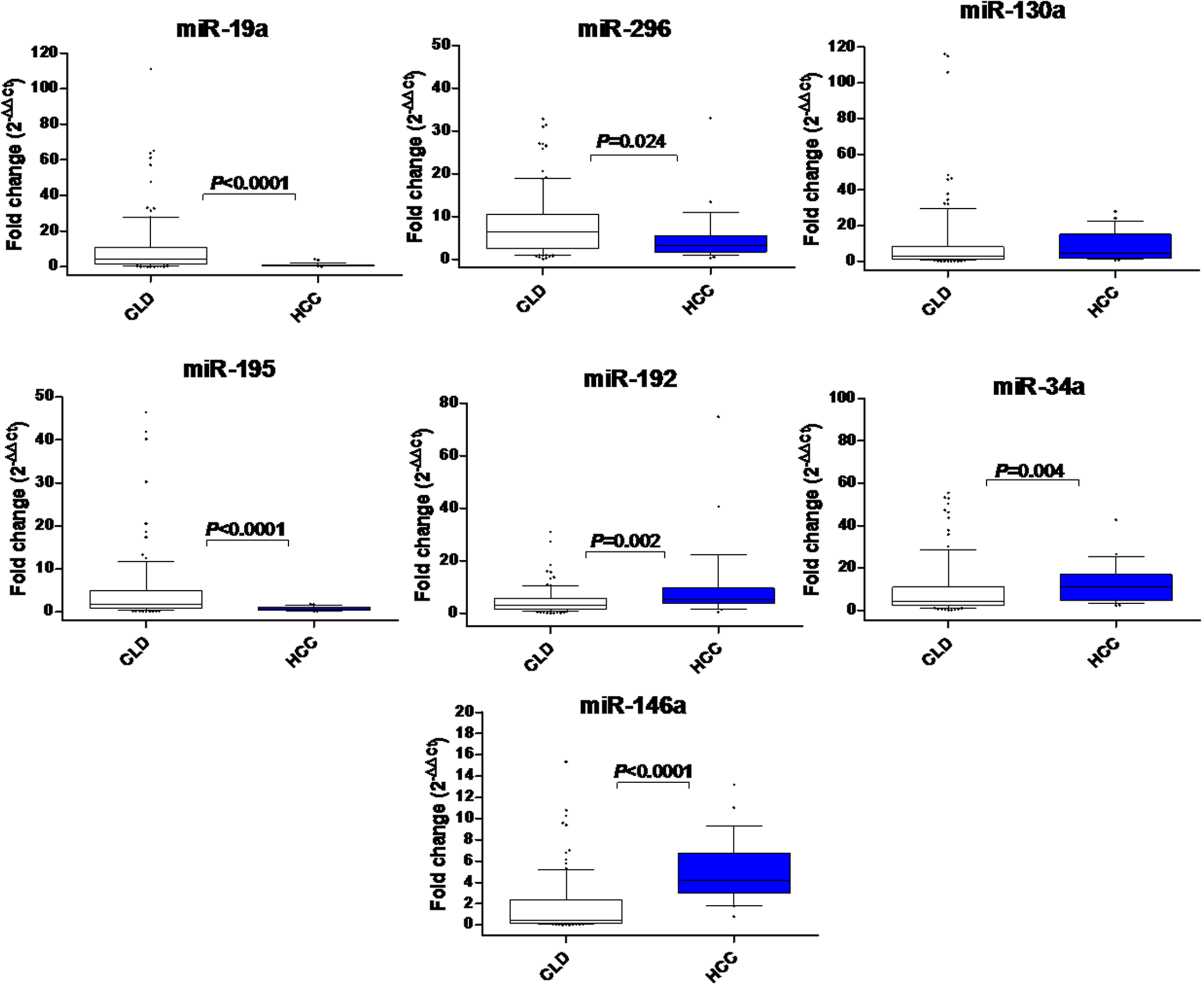
Differential expression of serum miRNA levels in HCC and CLD patients. The box represents the 25%-75% percentiles; the line inside the box represents the median and the upper and lower lines representing the 10%-90% percentiles of fold change in expression levels of studied miRNAs in serum of CLD (n = 125) and HCC patients (n = 112). Data were analyzed by Mann-Whitney *U*-test.

Comparing to CLD, miR-192, miR-34a, and miR-146a showed significant fold increase (*P* = 0.004, *P*<0.0001, and *P* = 0.002, respectively), whereas miR-19a, miR-296, and miR-195 showed significant fold decrease (*P*<0.0001, *P* = 0.024, and *P*<0.0001, respectively) in HCC. miR-130a relative expression was not significantly different between HCC and CLD (*P*>0.05) (Fig 2).

### Signature of serum miRNAs during HCV-related liver disease progression

**Fig 3:**
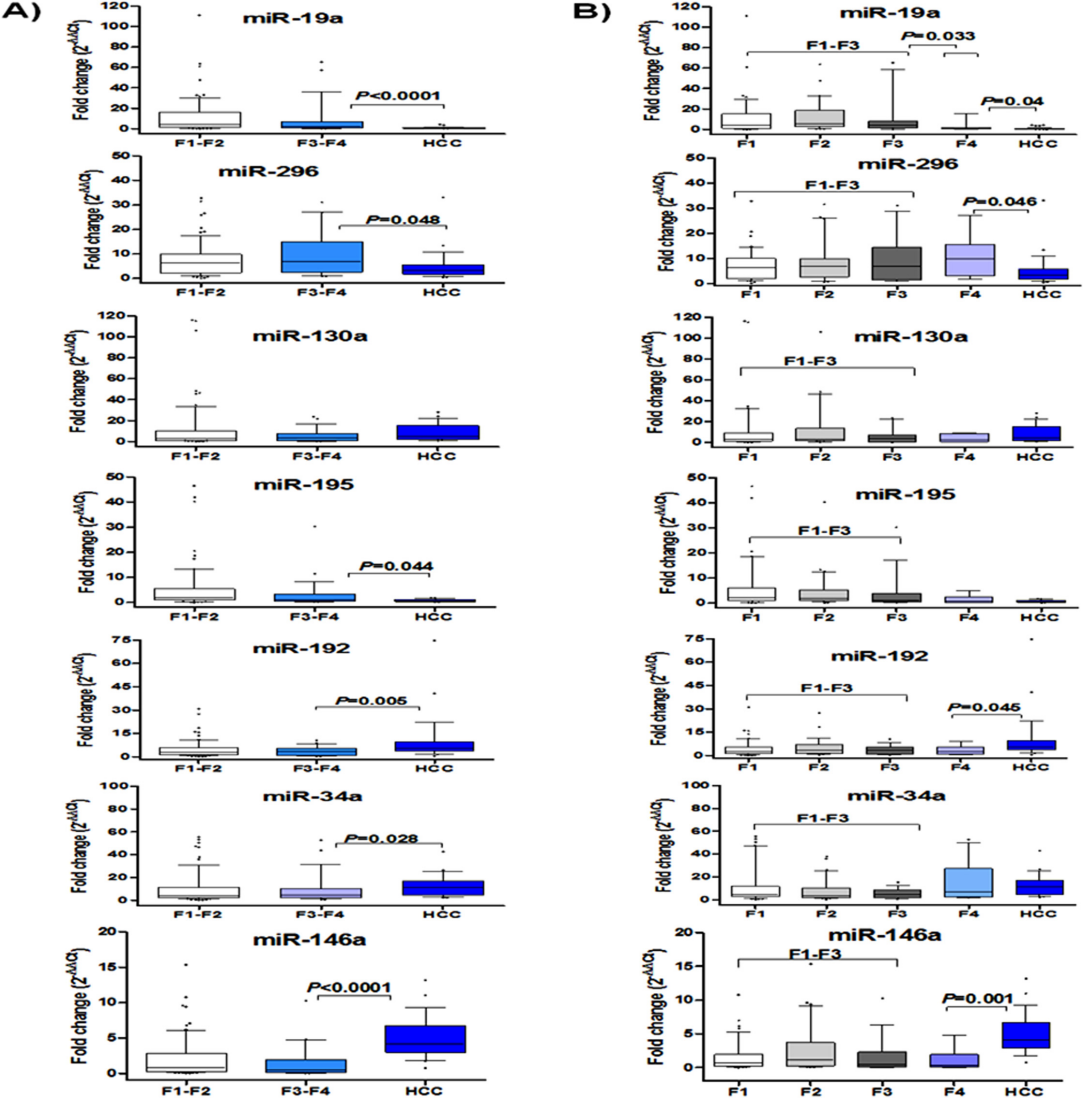
Signature of serum miRNAs relative expression during HCV-related liver disease progression. Panel (A) represents fold change of miRNA expression levels in early fibrosis (F1-F2, n = 75), late fibrosis (F3-F4, n = 50), and HCC (n = 112) groups. Comparison between F1-F2 vs F3-F4 or F3-F4 vs HCC was analyzed by Mann-Whitney *U*-test. Panel (B) represents detailed analysis of miRNA relative expression levels at different fibrosis stages (F1, n = 45, F2, n = 30, F3, n = 18, F4, n = 32) and HCC (n = 112). Comparison was done by Kruskal-Wallis test. Mann-Whitney *U*-test was used to compare F1-F3 vs F4 or F4 vs HCC. The box represents the 25%-75% percentiles; the line inside the box represents the median and the upper and lower lines representing the 10%-90% percentiles.

In a further analysis, we investigated serum miRNA profiles during the progression signature from F1-F2 to F3-F4 to HCC. We examined if serum miRNA levels would change from F1-F2 to F3-F4 or from F3-F4 to HCC using Mann-Whitney *U*-test. We found no significant difference in miRNA levels between F1-F2 and F3-F4, and except miR-130a, all studied miRNAs were significantly different when HCC and F3-F4 were compared (*P*<0.05) (Fig 3A). In a detailed analysis, we investigated serum miRNA levels in patients with individual fibrosis stages (F1 to F4) using the non-parametric Kruskal-Wallis test, with no significant difference was obtained (*P*>0.05) (Fig 3B). In a sub-analysis, we examined if studied miRNAs could be deregulated during progression from F1-F3 (liver fibrosis) to F4 (cirrhosis) or from F4 to HCC using Mann-Whitney *U*-test (Fig 3B). We found that miR-19a was significantly downregulated in F4 versus F1-F3 (*P* = 0.033). Moreover, miR-19a was further downregulated in HCC versus F4 (*P* = 0.04). In addition, miR-296, miR-192, and miR-146a levels were significantly different between HCC and F4 (*P*<0.05), however, they were not differentially expressed between F1-F3 versus F4. On the other hand, miR-195 and miR-34a were not significantly different neither for F1-F3 versus F4 nor for HCC versus F4 (*P*>0.05).

### Diagnostic performance of serum miRNAs

**Fig 4:**
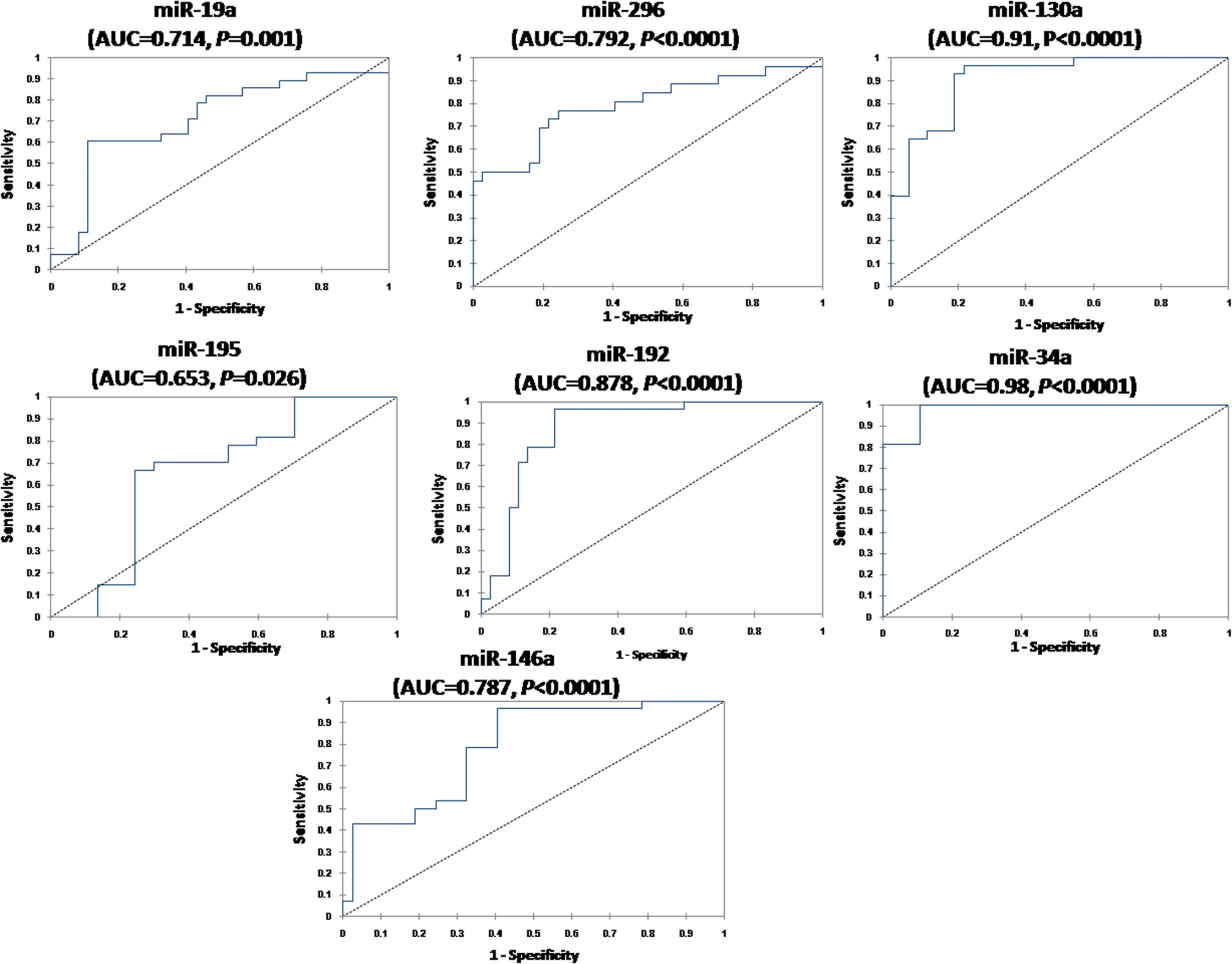
Serum miRNAs as diagnostic biomarkers to differentiate HCC patients from healthy controls. ROC curve analysis of serum miRNAs as diagnostic biomarkers differentiating HCC patients (n = 112) from healthy controls (n = 42).

ROC analysis revealed that studied miRNAs could discriminate between HCC and healthy controls (Fig 4) with AUC = 0.714 for miR-19a (95% CI 0.62–0.83, *P* = 0.001), 0.792 for miR-296 (95% CI 0.7–0.88, *P*<0.0001), 0.91 for miR-130a (95% CI 0.85–0.79, *P*<0.0001), 0.653 for miR-195 (95% CI 0.5–0.79, *P* = 0.045), 0.878 for miR-192 (95% CI 0.795–0.96, *P*<0.0001), 0.98 for miR-34a (95% CI 0.95–1, *P*<0.0001), and 0.787 for miR-146a (95% CI 0.69–0.88, *P*<0.0001), respectively.

**Fig 5:**
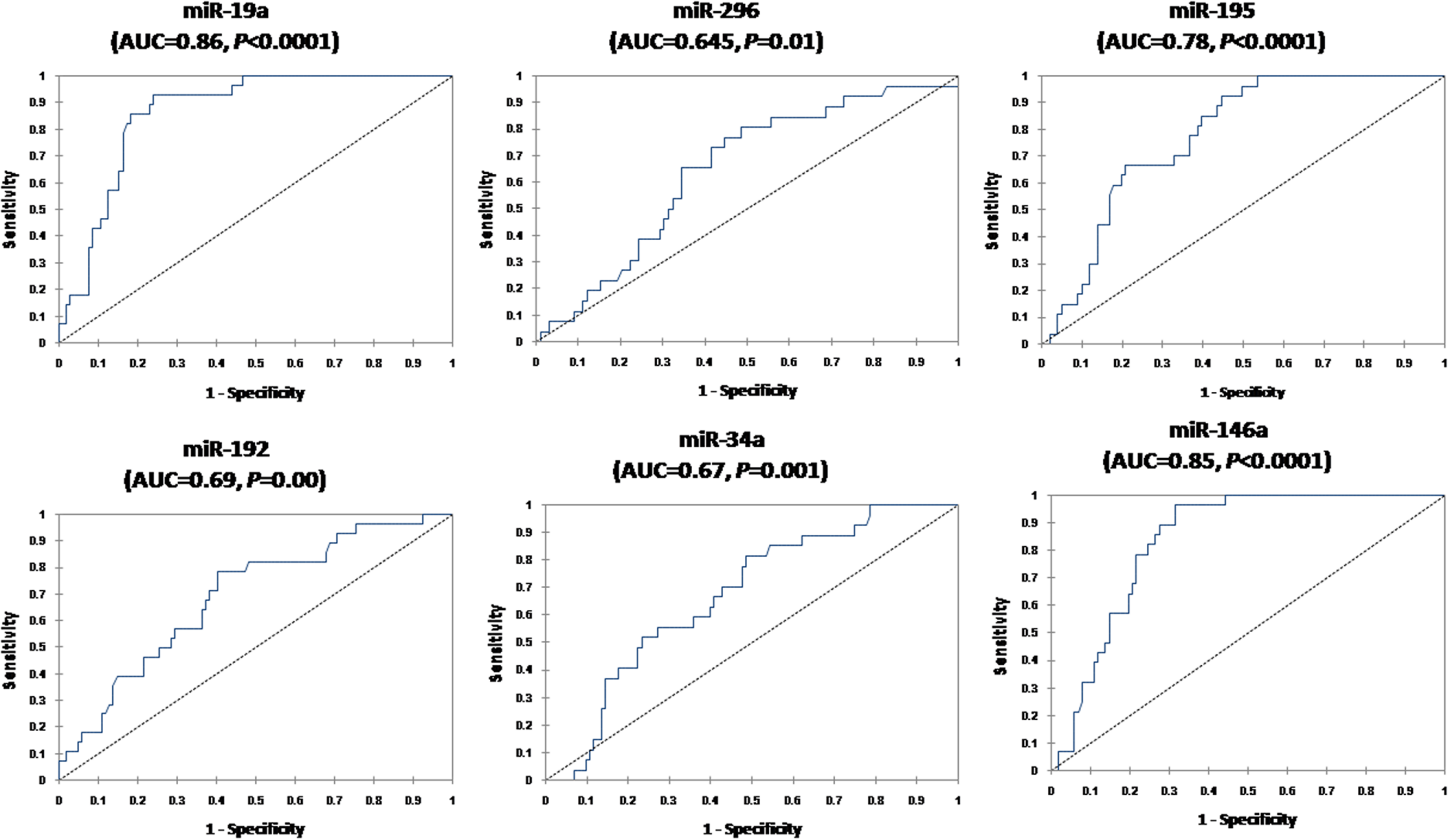
Serum miRNAs as diagnostic biomarkers to differentiate HCC from non-malignant CLD. ROC curve analysis of serum miRNAs as diagnostic biomarkers differentiating HCC patients (n = 112) from CLD (n = 125).

We examined the diagnostic performance of serum miR-19a, miR-296, miR-195, miR-192, miR-34a, and miR-146a which were differentially expressed in HCC and CLD to discriminate between the two groups. ROC analysis (Fig 5) revealed AUC = 0.86 (95% CI 0.79–0.92, *P*<0.0001) for miR-19a, 0.645 (95% CI 0.53–0.76, *P* = 0.01) for miR-296, 0.78 (95% CI 0.69–0.85, *P*<0.0001) for miR-195, 0.69 (95% CI 0.58–0.79, *P* = 0.00) for miR-192, 0.67(95% CI 0.58–0.78, *P* = 0.001) for miR-34a, and 0.85 (95% CI 0.88–0.97, *P*<0.0001) for miR-146a.

**Fig 6:**
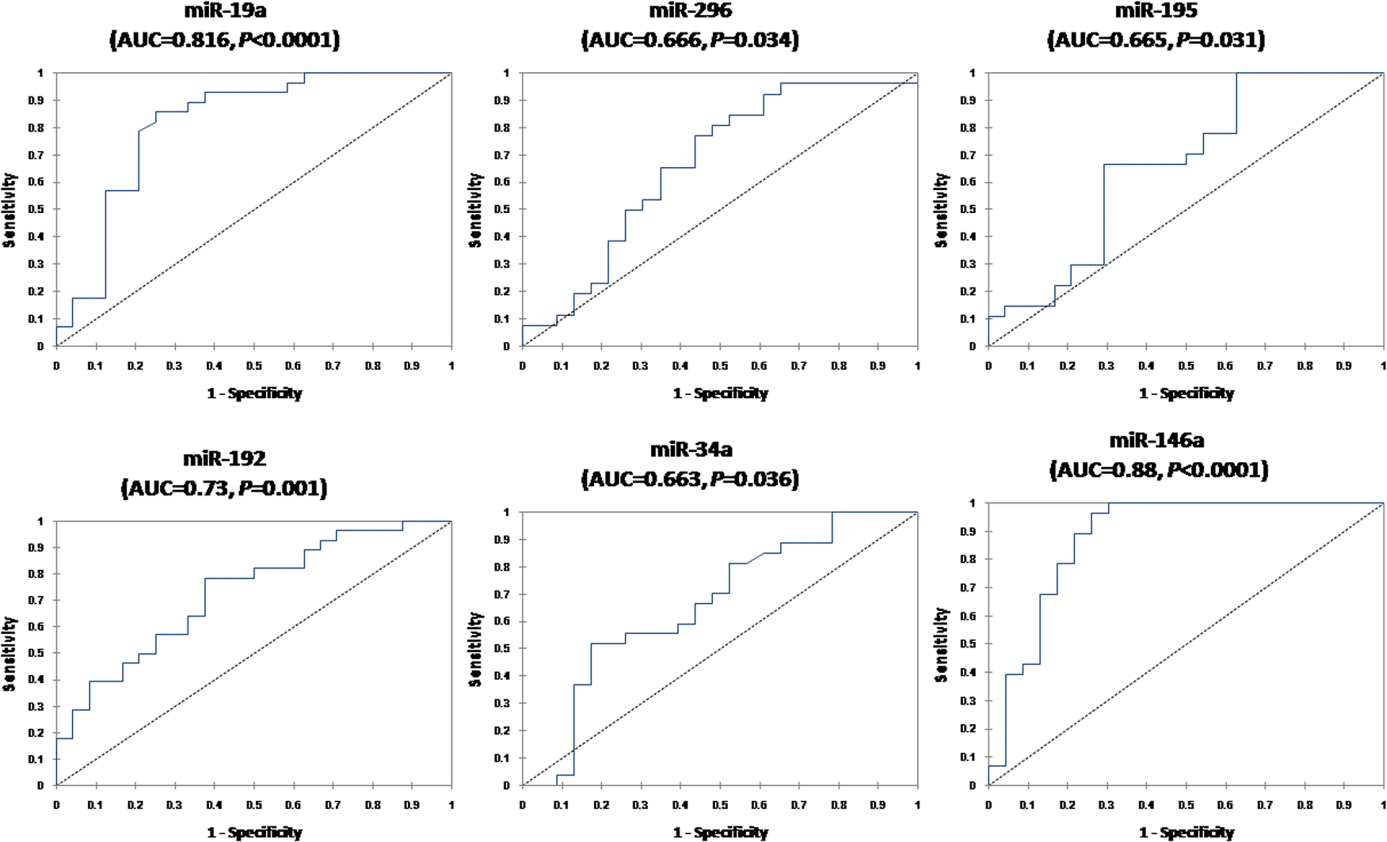
Serum miRNAs as diagnostic biomarkers to differentiate HCC patients from late fibrosis (F3-F4) subgroup. ROC curve analysis of serum miRNAs as diagnostic biomarkers differentiating HCC (n = 112) from F3-F4 patients (n = 50).

The end point is the identification of HCC patients versus late fibrosis. ROC curve results (Fig 6) revealed that these miRNAs could discriminate between HCC and F3-F4 with AUC = 0.816 for miR-19a (95% CI 0.69–0.94, *P*<0.0001), 0.666 for miR-296 (95% CI 0.51–0.82, *P* = 0.034), 0.665 for miR-195 (95% CI 0.51–0.82, *P* = 0.031), 0.73 for miR-192 (95% CI 0.59–0.86, *P* = 0.001), 0.663 for miR-34a (95% CI 0.53–0.82, *P* = 0.036), and 0.88 for miR-146a (95% CI 0.77–0.98,*P*<0.0001), respectively. The calculated sensitivities, specificities, and diagnostic accuracies for studied miRNAs to discriminate HCC from controls, CLD, and F3-F4 patients are shown in Table 3.

**Table 3:** Diagnostic performances of serum miRNAs to discriminate HCC patients from healthy controls, non-malignant CLD patients, and late fibrosis (F3-F4) subgroup.

miRNA	AUC	Best cutoff	Sensitivity	Specificity	PPV	NPV	Accuracy
		value (fold)	%	%	%	%	%
**miR-19a**							
HCC vs controls	0.714	<0.625	60.7	89.2	81	75	76.9
HCC vs CLD	0.86	<1.58	92.9	75.5	51	97.5	80
HCC vs F3-F4	0.816	<1.1	85.7	75	80	81.8	80.8
**miR-296**							
HCC vs controls	0.792	>1.5	76.9	75.7	69	82.4	76.2
HCC vs CLD	0.645	<5.49	76.9	55.6	31.3	90.2	60
HCC vs F3-F4	0.666	<5.83	76.9	56.5	66.7	68.4	67.3
**miR-130a**							
HCC vs controls	0.91	>1.05	96.4	78.4	77.1	96.7	86.2
**miR-195**							
HCC vs controls	0.653	<0.77	66.7	75.7	66.7	75.7	71.9
HCC vs CLD	0.78	<1.6	92.6	55.4	35.7	96.6	63.3
HCC vs F3-F4	0.665	<0.77	66.7	70.8	72	65.4	68.6
**miR-192**							
HCC vs controls	0.878	>1.35	96.4	78.4	77.1	96.7	86.2
HCC vs CLD	0.69	>3.52	78.6	59.8	34.9	91	63.8
HCC vs F3-F4	0.73	>3.42	78.6	62.5	71	71.4	71.2
**miR-34a**							
HCC vs controls	0.98	>1.5	100	89.2	87.1	100	93.8
HCC vs CLD	0.67	>4.18	81.5	51.5	30.6	91.4	57.7
HCC vs F3-F4	0.663	>10.88	51.9	82.6	77.8	59.4	66
**miR-146a**							
HCC vs controls	0.787	>1.4	96.4	59.5	63.4	95.7	75.4
HCC vs CLD	0.85	>1.5	96.4	69.3	46.6	98.6	75.2
HCC vs F3-F4	0.88	>0.889	96.4	73.9	81.8	94.4	86.3

PPV: Positive predictive value, NPV: negative predictive value.

### Logistic regression analysis of miRNAs

Univariate and multivariate logistic regression analyses were performed to select the predictor miRNAs associated with HCV-related HCC diagnosis (Table 4). Expression levels of miR-19a, miR-195, miR-192, and miR-146a were selected as significant predictors associated with the chances of HCC diagnosis in the univariate analysis. In a stepwise forward multivariate analysis, these four miRNAs turned out to be significant predictors of the risk of being diagnosed with HCC. The predicted probability of being diagnosed with HCC from the logit model based on the 4-miRNA panel (Table 4), Logit(P) = -0.24–1.114*mir-19a-0.077*miR-195+0.571*miR-192+0.048*miR-146a, was used to construct the ROC curve.

**Table 4:** Logistic regression analysis of miRNAs.

Parameter	Coefficient	SE	P value	Odds ratio	Odds ratio (95% CI)
**Univariate analysis**					
miR-19a	-0.56	0.107	**<0.0001**	0.571	0.463–0.705
miR-296	-0.006	0.026	0.825	0.994	0.945–1.046
miR-195	-0.066	0.014	**<0.0001**	0.936	0.91–0.963
miR-192	0.186	0.045	**<0.0001**	1.204	1.101–1.318
miR-34a	0.002	0.012	0.844	1.002	0.98–1.025
miR-146a	0.068	0.01	**<0.0001**	1.07	1.05–1.09
**Multivariate analysis**					
miR-19a	-1.114	0.224	**<0.0001**	0.328	0.212–0.51
miR-195	-0.077	0.027	**0.004**	0.926	0.878–0.976
miR-192	0.571	0.119	**<0.0001**	1.77	1.4–2.23
miR-146a	0.048	0.015	**0.002**	1.049	1.018–1.08
Constant	-0.24				

A stepwise forward multivariate analysis including miR-19a, miR-195, miR-192, and miR-146a was conducted with a probability of entry *P<*0.05 and probability of removal *P*>0.1. Overall model fit: Null model -2 Log Likelihood = 375.86; Full model -2 Log Likelihood = 175.41; X^2^ = 202.44, *P*<0.0001. Logit(P) = -0.24–1.114*mir-19a-0.077*miR-195+0.571*miR-192+0.048*miR-146a; AUC = 0.946. SE: standard error, *P* values in bold are statistically significant (*P*<0.05).

**Fig 7:**
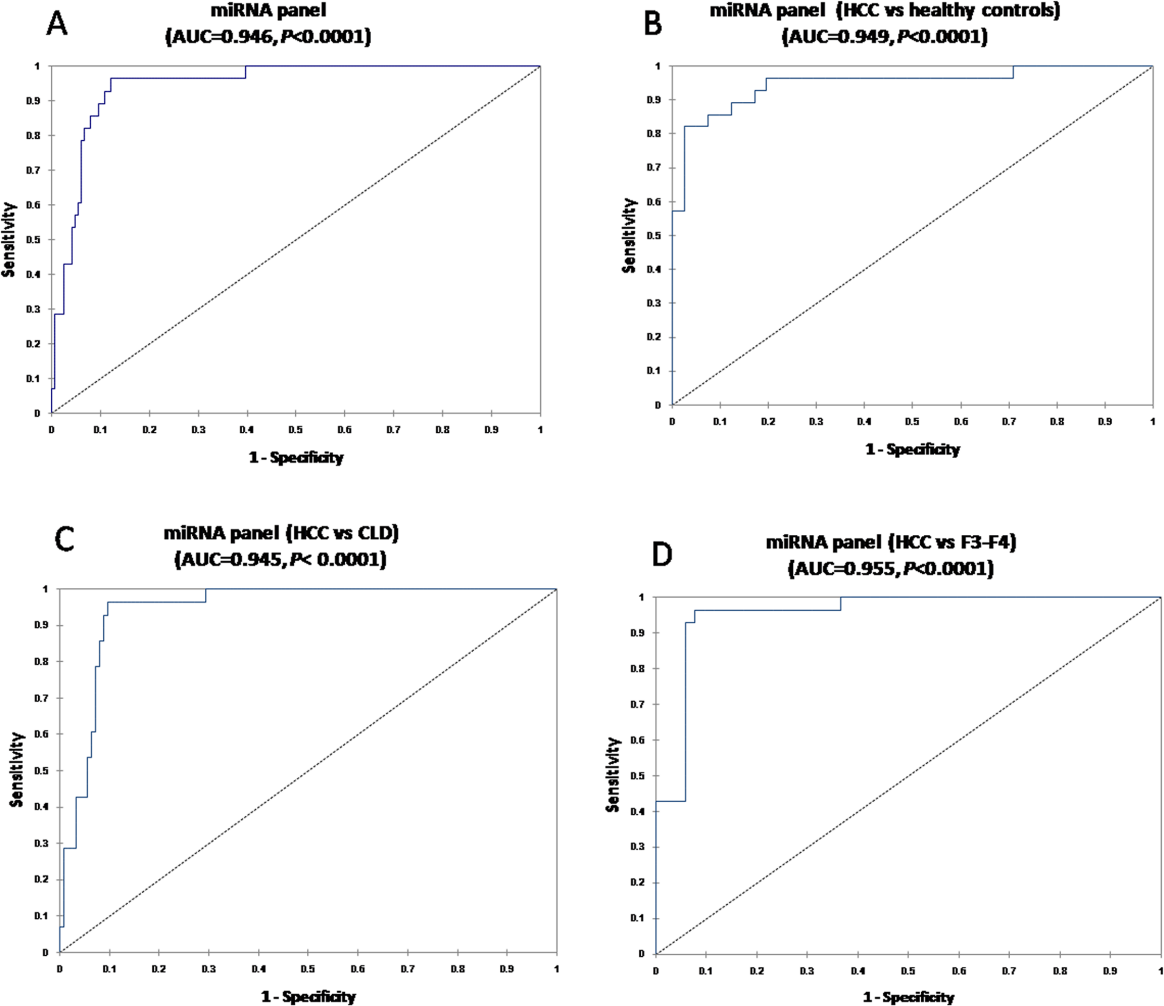
Diagnostic performance of the miRNA panel. AUCs of the miRNA panel (A) and miRNA panel in differentiating HCC from healthy controls (B), CLD (C), and F3-F4 patients (D).

The diagnostic performance for the established miRNA panel was evaluated using ROC analysis. The AUC for the miRNA panel was 0.946 (95% CI = 0.912–0.977) with sensitivity = 96.2%, specificity = 88%, and diagnostic accuracy = 91.4% (Fig 7A). The miRNA panel could also discriminate HCC from healthy controls (AUC = 0.949; 95% CI, 0.9–0.98; sensitivity = 82.1%; specificity = 97.6%; diagnostic accuracy = 86.3%) (Fig 7B), CLD (AUC = 0.945; 95% CI, 0.915–0.975; sensitivity = 96.4%; specificity = 90.5%; diagnostic accuracy = 93.3%) (Fig 7C), and F3-F4 subgroup (AUC = 0.955; 95% CI, 0.919–0.991; sensitivity = 96.4%; specificity = 92.3%; accuracy = 95.1%) (Fig 7D). Comparison of the AUC of the miRNA panel with that of individual miRNAs revealed that the miRNA panel was superior to individual miRNAs in discriminating HCC from healthy controls (except for miR-34a and miR-130a) (S1 Table), CLD (S2 Table) and F3-F4 (S3 Table) patients. The results indicate that the miRNA panel has a higher sensitivity and specificity for HCC detection especially in at high-risk patients.

### Comparison of the AUC of the miRNA panel with that of AFP

**Fig 8:**
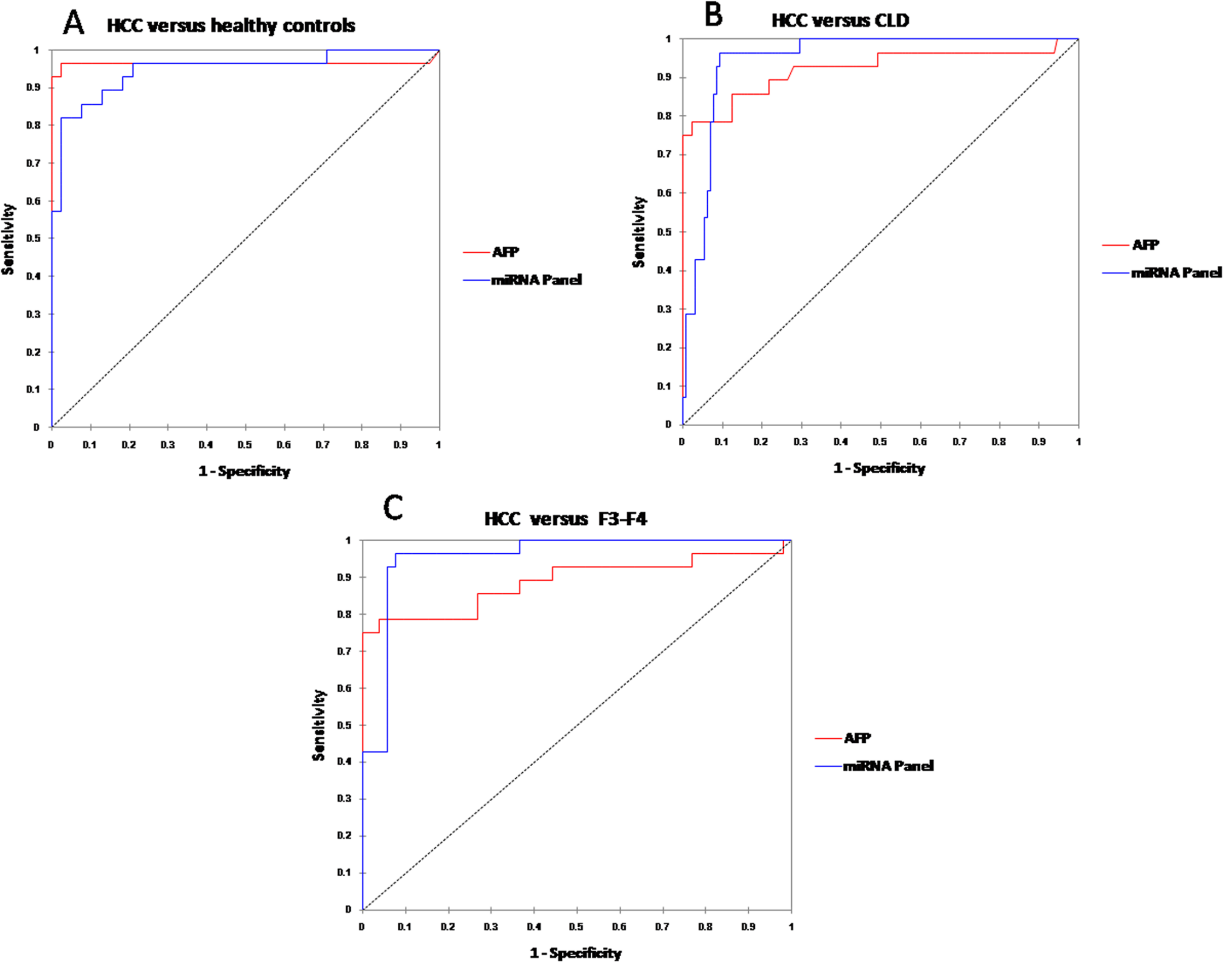
Comparison of AUC of the miRNA panel with that of AFP. AUCs of the miRNA panel and AFP in differentiating HCC from healthy controls (A), CLD (B), and F3-F4 patients (C).

Using the same serum samples, the AUC of AFP in different groups was evaluated. AFP demonstrated high accuracy in discriminating HCC from healthy controls (AUC = 0.963; 95% CI, 0.93–0.99), CLD patients (AUC = 0.921; 95% CI, 0.884–0.95), and F3-F4 subjects (AUC = 0.888; 95% CI, 0.84–0.936). At cutoff >20 ng/ml, AFP has 100%, 96.12%, and 91% specificity and 75% sensitivity to differentiate HCC from healthy controls, CLD, and F3-F4 subjects, respectively. However, AFP >400 ng/ml is considered diagnostic for HCC, although 46% of our patients reported levels that high. At this cutoff, AFP has 100%, 97%, and 95% specificity and 46% sensitivity to differentiate HCC from controls, CLD, and F3-F4 subgroup, respectively. We also compared the AUC of the miRNA panel with that of AFP. There was no difference between the AUC values of the miRNA panel and those of AFP (difference between areas = 0.018, *P* = 0.503) in the healthy group (Fig 8A) and CLD (difference between areas = 0.024, *P* = 0.348) (Fig 8B). However, there was significant difference between the AUC values of the miRNA panel and those of AFP in F3-F4 subgroup (difference between areas = 0.067, *P* = 0.041) (Fig 8C).

### Correlations between studied serum miRNAs in HCC group

**Fig 9:**
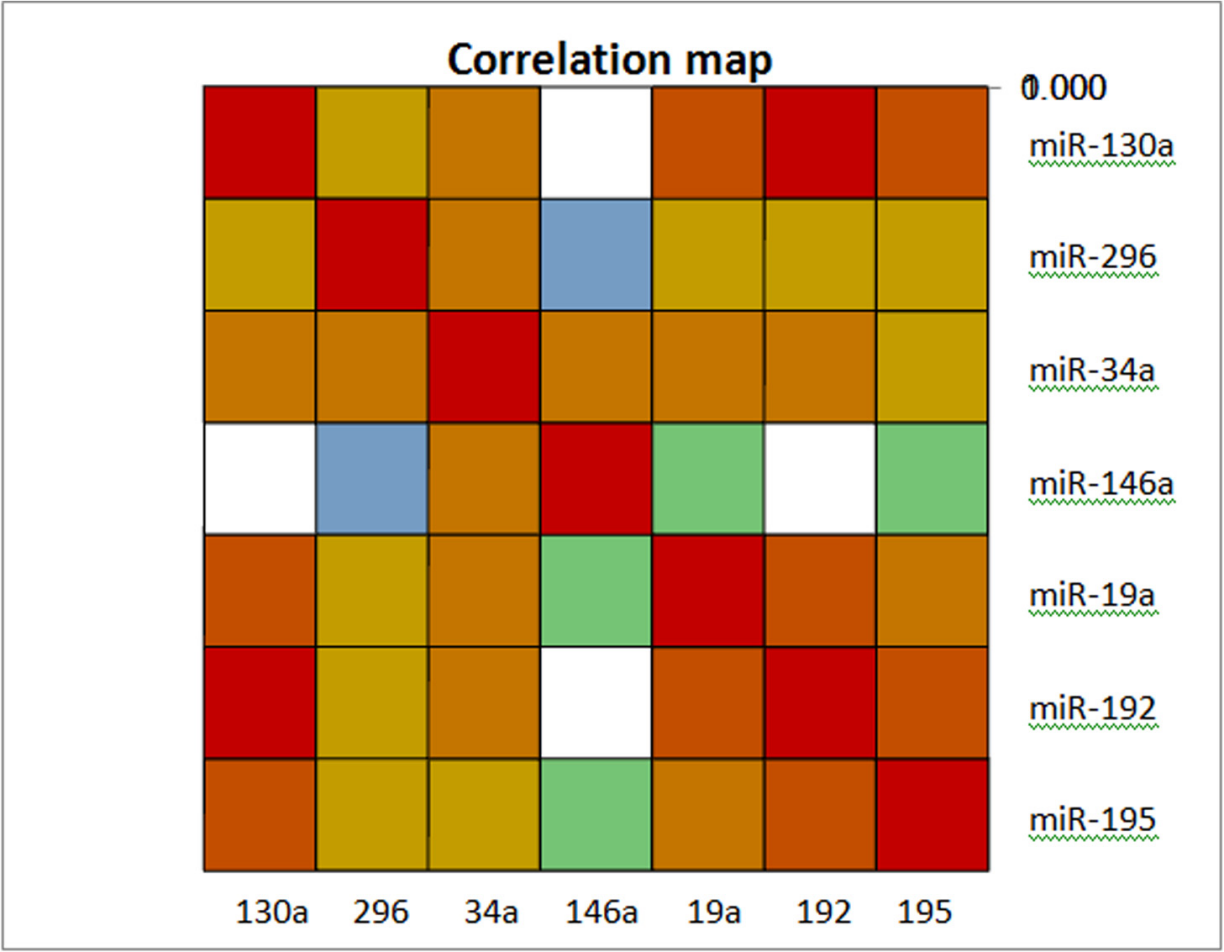
Correlations between serum miRNAs levels in HCC group. A correlation map with a blue-red (cold-hot) scale. The blue color corresponds to a correlation close to -1 and the red color corresponds to a correlation close to 1. Green corresponds to a correlation close to 0. Correlations are made by spearman correlation.

We found several significant positive correlations between studied miRNAs in the HCC group (Fig 9, S4 Table). miR-19a was correlated with miR-130a (Spearman r = 0.657, *P*<0.0001), miR-195 (r = 0.522, *P* = 0.006), miR-192 (r = 0.619, *P* = 0.0004), and miR-34a (r = 0.451, *P* = 0.018). miR-296 was correlated with miR-130a (r = 0.467, *P* = 0.016), miR-192 (r = 0.408, *P* = 0.038), and miR-34a (r = 0.543, *P* = 0.005). miR-130a was also correlated with miR-195 (r = 0.676, *P*<0.0001), miR-192 (r = 0.857, *P*<0.0001), and miR-34a (r = 0.516, *P* = 0.005). miR-192 was also correlated with miR-195 (r = 0.597, *P* = 0.001) and miR-34a (r = 0.451, *P* = 0.018). miR-34a was correlated with miR-146a (r = 0.41, *P* = 0.033).

### Correlations between miRNAs and clinicopathological data in HCC group

miR-19a was significantly higher in HCC patients with PV thrombosis (n = 24) than those without [median fold change (25%-75% percentiles), 0.98 (0.88–1.37) versus 0.38 (0.2–0.69), respectively, *P* = 0.0003]. No correlations were found between studied miRNAs and number of focal lesions, diameter of focal lesions, tumor size, AFP or any other clinical data. miR-34a was positively correlated with child stage (Pearson r = 0.574, *P* = 0.001) and BCLC score (Pearson r = 0.487, *P* = 0.01).

## Discussion

Early diagnosis of HCC presents a challenge due to lack of reliable biomarkers, hence stressing the need for new early diagnostic tools. The present study revealed that all studied miRNAs were differentially expressed in serum of HCV-related HCC patients versus controls, and except miR-130a, they were differentially expressed between HCC and non-malignant CLD, implicating these miRNAs as surrogate biomarkers of HCC. In addition, studied miRNAs distinguished HCC from controls, and except miR-130a, they distinguished HCC from CLD, and in particular from late fibrosis subgroup. Multivariate analysis revealed a serum miRNA panel of miR-19a, miR-146a, miR-195, and miR-192 with high diagnostic accuracy for HCC diagnosis. Moreover, the miRNA panel demonstrated significantly higher diagnostic accuracy than AFP in late fibrosis patients, which at much high-risk to develop HCV-related HCC. However, this panel has higher diagnostic accuracy than previously reported for AFP (sensitivity:39–65%; specificity: 76–94%) [9]. Nearly one third of early HCC patients are missed by AFP analysis and it is also elevated in patients with chronic hepatitis and cirrhosis [9]. These results implicate these miRNAs as reliable early biomarkers and possible therapeutic tools or targets for HCC treatment. Perhaps addition of this miRNA panel to serological tumor markers may improve the diagnostic accuracy for early HCC detection.

The current study revealed that the tested miRNAs were positively correlated in HCC group, suggesting their concordant deregulation in HCC. We have previously shown that serum expression levels of these miRNAs were correlated in chronic HCV patients and perhaps implicated in HCV pathogenesis [32]. Together, these results may relate at least some of the investigated miRNAs to the hepatocarcinogenic effect of HCV, which need further investigation. Notably, HCV proteins were shown to affect apoptosis and tumor cell behavior by altering miRNAs expression in HCC cells expressing full length-HCV [8].

Serum miR-19a and miR-195 were downregulated in HCC versus controls or CLD patients. Similarly, serum miR-19a was downregulated in HCC versus chronic HBV [33]. miR-195 was also downregulated in serum of HCC patients [37] and in HCC tissues versus healthy livers [27,28,38]. Downregulation of miR-195 promotes carcinogenesis by targeting FGF7 and GHR, both induce evading apoptosis, tissue invasion, and metastasis [6]. The present study also demonstrated that higher levels of serum miR-19a were associated with PV thrombosis in HCC patients, implying a role of miR-19a in HCC invasion and progression. miR-19a was also associated with HCC satellite nodules [39], HCC recurrence and predicted survival after liver transplantation [21]. Together, these results may emphasize the prognostic value of miR-19a in HCC.

Serum miR-296 and miR-130a were upregulated in HCC versus controls. miR-296 was significantly lower in HCC versus CLD while miR-130a was not changed. In contrast, miR-296 [26] and miR-130a [27,28] were downregulated in HCC tissues versus healthy livers, with miR-296 was not different between HCC and HCV-infected liver tissues [26], while miR-130a was differentially expressed between HCC and chronic hepatitis liver tissues [19]. Serum miR-192, miR-146a, and miR-34a were upregulated in HCC versus controls or CLD in this study. Similarly, serum miR-192 and miR-146a were upregulated in HCC or liver cirrhosis [33,40]. miR-34a was also upregulated in HCC tissues versus healthy livers [29–31]. In contrast, miR-34a was downregulated in human cancers, including HCC [41,42]. Serum miR-192 was also downregulated in HCC versus controls but not changed relative to cirrhosis [43], with miR-146a being downregulated in HCC tissues versus healthy livers or adjacent non-cancerous tissue [27,28,44–46]. These conflicting results may reflect that the same miRNA may have dual tumor suppressor or oncogenic roles in cancer. Interestingly, while miR-296 was downregulated during tumor progression in correlation with metastasis in many cancers, including HCC [47], miR-296 promoted tumor angiogenesis in endothelial cells [48]. miR-146a also suppresses HCC invasion or metastasis via downregulating VEGF [46], and its downregulation was correlated with HCC deterioration [45]. Conversely, miR-146a was upregulated in HCC cells and exerted negative effects on anti-tumor immune response [49].

In the current study, serum levels of 2 out of 6 studied miRNAs were matched with hepatic tissue levels in HCC versus controls [27–29,31], however, to the best of our knowledge, miR-19a levels in HCC tissues versus normal liver are not known. Circulating and tissue miRNA levels are not always consistent; miR-122 was downregulated in HCC tissues and cancer cell lines [50], but upregulated in serum of HCC patients [51,52]. This inverse relationship suggests that secreted miRNAs from cells may be an important component of circulating miRNA expression [9]. The liver secretes circulating exosomes during injury with increase in serum miR-122 and miR-192, and a corresponding decrease in their hepatic expression [53]. On the other hand, it was reported that miRNAs that were elevated in the liver during injury displayed decreased levels in serum [54]. The variable levels of miRNAs in HCC between different studies may also reflect the counter mechanisms that regulate their expression. For example, miR-34a is activated by p53 and inactivated by DNA methylation; liver tumors that still retain an active p53 may upregulate miR-34a [31]. miR-146a is downregulated by DNA methylation [46] and upregulated by aberrantly activated STAT3 in HCC cells [49]. The discrepancies between different studies may also arise from variability in technical procedures from sampling to detection method and data analysis, or the use of different normalization controls or control tissues used for normalization (healthy liver or adjacent non-tumor tissue) [55]. HCC etiology should also be considered, for example, HBV-HCC and HCV-HCC exhibited different miRNA dysregulation patterns and distinct molecular mechanisms [19].

Progression towards HCC involves multiple steps and the clinical pathway of most chronic HCV cases comprises fibrosis progression to cirrhosis, and eventually HCC [11]. We demonstrated reduced hepatic synthetic function (albumin and prothrombin) during liver disease progression among studied groups, but neither was correlated with miRNAs. Hepatic miRNA expression is deregulated in liver fibrosis, and thus circulating miRNAs are expected to be affected by fibrosis progression [13]. miR-19a was upregulated in CLD patients versus controls (4.5 fold) and detailed analysis of its levels within CLD group revealed reduced levels during progression from liver fibrosis to cirrhosis, then further downregulation in HCC. Serum miRNA levels may initially rise following release from inflamed hepatocytes in HCV followed by a drop in the levels with fibrosis progression due to hepatocyte loss and accumulation of extracellular matrix [56]. Activation of hepatic stellate cells, a key driver of fibrosis, is also associated with a specific miRNA deregulation regulating various fibrogenic signaling pathways [57]. It seems probable that miR-19a may be implicated in inflammatory and fibrogenic processes in CLD progression, ultimately to HCC. Other studied miRNAs were fibrosis-stage independent and differential expression only occurs from late fibrosis to HCC. Conversely, miR-34a was overexpressed during progression from normal liver through cirrhosis to HCC [58], with serum miR-34a being increased during fibrosis progression in chronic HCV [13]. These conflicting results may render miR-34a an inappropriate marker, when exclusively used for interpretation of fibrosis progression. We demonstrated that serum miR-34a was positively correlated with child stage and BCLC score, a staging system which defines HCC patients for different treatment strategies. This correlation may delineate the usefulness of serum miR-34a in assessing HCC severity, staging, and patient classification for therapeutic interventions.

Previous studies have evaluated circulating miRNAs as HCC biomarkers in comparison to healthy controls and chronic hepatitis or cirrhosis [35,37,40,43,51,59,60], with few miRNAs revealed as candidate biomarkers. Our study revealed a four miRNA set with high accuracy for HCC and could be of clinical value in diagnosis of HCV-related HCC. In addition, most of these studies have addressed HCC of HBV etiology or mixed etiologies, including HCV, with few solely studied HCV-related HCC. Another limitation is that neither study has evaluated circulating miRNAs as potential biomarkers during CLD progression to HCC. Our study is unique as we demonstrated serum miRNA signatures during stepwise progression form liver fibrosis to HCC. We hypothesized that the miRNA that changes significantly from early fibrosis to late fibrosis to HCC would be a biomarker of disease progression. Perhaps our prominent result is that miR-19a levels were negatively associated with fibrosis progression to cirrhosis, finally to HCC. However, our study is limited by the relatively small number of patients and additional studies are required to validate the miRNA panel in an independent larger patient cohort and test its prognostic significance in HCC outcome and the implications of these miRNAs as therapeutic tools or targets in HCC treatment. Longitudinal samples should be also considered in monitoring liver fibrosis progression, eventually to HCC.

## Conclusion

Studied miRNAs, but not miR-130a, could distinguish HCV-related HCC from HCV-associated CLD and in particular, from late fibrosis (F3-F4) subgroup, suggesting the potential usefulness of these miRNAs as early biomarkers of HCC detection in high-risk groups. We identified a serum miRNA panel of miR-19a, miR-146a, miR-192, and miR-195 with high accuracy for HCC and could be of clinical value in diagnosis of HCV-related HCC. Additional studies are needed to confirm this panel in a larger patient cohort and test its prognostic significance. Serum miRNAs were fibrosis-stage independent, however, miR-19a was significantly downregulated during disease progression from liver fibrosis (F1-F3) to cirrhosis (F4) to HCC, suggesting that serum miR-19a monitoring could be of clinical relevance as a potential diagnosis tool of fibrosis progression to HCC. The investigated miRNAs were significantly correlated in HCC group, suggesting their concordant deregulation in HCC. The correlations between miR-19a and miR-34a with PV thrombosis and BCLC staging scores, respectively, may emphasize their prognostic significance in HCC.

## Supporting Information

S1 Table Comparison of ROC curves between the miRNA panel and individual miRNAs in HCC versus healthy controls. (DOCX)

S2 Table Comparison of ROC curves between the miRNA panel and individual miRNAs in HCC versus CLD. (DOCX)

S3 Table Comparison of ROC curves between the miRNA panel and individual miRNAs in HCC versus F3-F4 subgroup. (DOCX)

S4 Table Significant correlations between studied miRNAs in HCC group. (DOCX)
