# Supplementary material for: Serum MicroRNAs as Potential Biomarkers for Early Diagnosis of Hepatitis C Virus-Related Hepatocellular Carcinoma in Egyptian Patients
Source: PLoS One. 2015 Sep 9;10(9):e0137706. doi: 10.1371/journal.pone.0137706 (PMC4564244; doi:10.1371/journal.pone.0137706)
Supplement: S1 Table — (DOCX) [file pone.0137706.s001.docx]

Table S1 Comparison of ROC curves between miRNAs panel and miRNAs in HCC versus healthy controls.

|  | | | | |
| --- | --- | --- | --- | --- |
| Variable | AUC | 95% CI | z statistic | *P* |
| miR-19a^a^ | 0.714 | 0.62–0.83 | 5.026 | <0.0001 |
| miR-296^b^ | 0.792 | 0.7–0.88 | 3.86 | 0.0001 |
| miR-130a^c^ | 0.91 | 0.85–0.79 | 1.379 | 0.167 |
| miR-195^d^ | 0.653 | 0.5–0.79 | 5.861 | <0.0001 |
| miR-192^e^ | 0.878 | 0.795–0.96 | 2.22 | 0.026 |
| miR-34a^f^ | 0.98 | 0.95–1 | -1.606 | 0.108 |
| miR-146a^g^ | 0.787 | 0.69–0.88 | 3.948 | <0.0001 |
| miRNA panel | 0.949 | 0.9-0.98 |  |  |
| Pairwise comparison, ^a^miRNA panel & miR-19a; ^b^miRNA panel & miR-296; ^c^miRNA panel & miR-130a; ^d^miRNA panel & miR-195; ^e^miRNA panel & miR-192; ^f^miRNA panel & miR-34a; ^g^miRNA panel & miR-146a. | | | | |
