# Supplementary material for: Serum MicroRNAs as Potential Biomarkers for Early Diagnosis of Hepatitis C Virus-Related Hepatocellular Carcinoma in Egyptian Patients
Source: PLoS One. 2015 Sep 9;10(9):e0137706. doi: 10.1371/journal.pone.0137706 (PMC4564244; doi:10.1371/journal.pone.0137706)
Supplement: S2 Table — (DOCX) [file pone.0137706.s002.docx]

Table S2 Comparison of ROC curves between miRNAs panel and miRNAs in HCC versus CLD.

|  | | | | |
| --- | --- | --- | --- | --- |
| Variable | AUC | 95% CI | z statistic | *P* |
| miR-19a^a^ | 0.86 | 0.79–0.92 | 2.84 | 0.004 |
| miR-296^b^ | 0.645 | 0.53–0.76 | 7.436 | <0.0001 |
| miR-195^c^ | 0.78 | 0.69–0.85 | 4.702 | <0.0001 |
| miR-192^d^ | 0.69 | 0.58–0.79 | 6.547 | <0.0001 |
| miR-34a^e^ | 0.67 | 0.58–0.78 | 6.942 | <0.0001 |
| miR-146a^f^ | 0.85 | 0.88–0.97 | 3.102 | 0.0002 |
| miRNA panel | 0.945 | 0.915-0.975 |  |  |
| Pairwise comparison, ^a^miRNA panel & miR-19a; ^b^miRNA panel & miR-296; ^c^miRNA panel & miR-195; ^d^miRNA panel & miR-192; ^e^miRNA panel & miR-34a; ^f^miRNA panel & miR-146a. | | | | |
