# Supplementary material for: Serum MicroRNAs as Potential Biomarkers for Early Diagnosis of Hepatitis C Virus-Related Hepatocellular Carcinoma in Egyptian Patients
Source: PLoS One. 2015 Sep 9;10(9):e0137706. doi: 10.1371/journal.pone.0137706 (PMC4564244; doi:10.1371/journal.pone.0137706)
Supplement: S3 Table — (DOCX) [file pone.0137706.s003.docx]

Table S3 Comparison of ROC curves between miRNAs panel and miRNAs in HCC versus F3-F4 subgroup.

|  | | | | |
| --- | --- | --- | --- | --- |
| Variable | AUC | 95% CI | z statistic | p |
| miR-19a^a^ | 0.816 | 0.69–0.94 | 3.843 | 0.0001 |
| miR-296^b^ | 0.666 | 0.51–0.82 | 6.233 | <0.0001 |
| miR-195^c^ | 0.665 | 0.51–0.82 | 6.247 | <0.0001 |
| miR-192^d^ | 0.73 | 0.59–0.86 | 5.271 | <0.0001 |
| miR-34a^e^ | 0.663 | 0.53–0.82 | 6.277 | <0.0001 |
| miR-146a^f^ | 0.88 | 0.77–0.98 | 2.944 | 0.012 |
| miRNA panel | 0.955 | 0.919-0.991 |  |  |
| Pairwise comparison, ^a^miRNA panel & miR-19a; ^b^miRNA panel & miR-296; ^c^miRNA panel & miR-195; ^d^miRNA panel & miR-192; ^e^miRNA panel & miR-34a; ^f^miRNA panel & miR-146a. | | | | |
