# Supplementary material for: Serum MicroRNAs as Potential Biomarkers for Early Diagnosis of Hepatitis C Virus-Related Hepatocellular Carcinoma in Egyptian Patients
Source: PLoS One. 2015 Sep 9;10(9):e0137706. doi: 10.1371/journal.pone.0137706 (PMC4564244; doi:10.1371/journal.pone.0137706)
Supplement: S4 Table — (DOCX) [file pone.0137706.s004.docx]

Table S4 Significant correlations between studied miRNAs in HCC group.

| miRNA | miR-130a | miR-296 | miR-34a | miR-146a | miR-19a | miR-192 | miR-195 |
| --- | --- | --- | --- | --- | --- | --- | --- |
| miR-130a | - | r=0.467 | r=0.516 | NS | r=0.657 | r=0.857 | r=0.676 |
|  |  | *P*=0.016 | *P*=0.005 |  | *P*<0.0001 | *P*<0.0001 | *P*<0.0001 |
| miR-296 | r=0.467 | - | r=0.543 | NS | NS | r=0.408 | NS |
|  | *P*=0.016 |  | *P*=0.005 |  |  | *P*=0.038 |  |
| miR-34a | r=0.516 | r=0.543 | - | r=0.41 | r=0.451 | r=0.451 | NS |
|  | *P*=0.005 | *P*=0.005 |  | *P*=0.033 | *P*=0.018 | *P*=0.018 |  |
| miR-146a | NS | NS | r=0.41 | - | NS | NS | NS |
|  |  |  | *P*=0.033 |  |  |  |  |
| miR-19a | r=0.657 | NS | r=0.451 | NS | - | r=0.619 | r=0.522 |
|  | *P*<0.0001 |  | *P*=0.018 |  |  | *P*=0.0004 | *P*=0.006 |
| miR-192 | r=0.857 | r=0.408 | r=0.451 | NS | r=0.619 | - | r=0.597 |
|  | *P*<0.0001 | *P*=0.038 | *P*=0.018 |  | *P*=0.0004 | r=0.451 | *P*=0.001 |
| miR-195 | r=0.676 | NS | NS | NS | r=0.522 | r=0.597 | - |
|  | *P*<0.0001 |  |  |  | *P*=0.006 | *P*=0.001 |  |

r:Spearman rho coefficient, NS: non-significant
